# Practical guide to structuring the Background section in clinical and epidemiological studies

**DOI:** 10.1186/s12998-026-00643-1

**Published:** 2026-09-01

**Authors:** Charlotte Leboeuf-Yde, Claire Gudex

**Affiliations:** 1 Department of Regional Health Research, University of Southern Denmark, Odense, Denmark; 2 Research Unit OPEN, Department of Clinical Research, University of Southern Denmark, Odense, Denmark

**Keywords:** Writing process, Structure of a scientific article, Background section, Research objectives

## Abstract

This is the first of three articles that provide step-by-step guides on how to structure the content of a scientific article to ensure a coherent and relevant text. This article deals with the Background section, with extra focus on the research objectives. We introduce the four typical elements of a Background section: (i) justification of the study topic, (ii) sufficient information for the reader to understand the research project, (iii) presentation of the research gaps that justify the research objectives, and (iv) well-constructed research objectives that indicate the study design and may include a purpose statement. Because the whole text is driven by the research objectives, we first suggest some tips for making these as informative as possible. We then provide a step-by-step approach for planning the structure and contents of the Background section as preparation for writing the full text later. We illustrate our explanations with text examples selected from this journal to show how authors have approached crucial aspects of the Background section. Finally, we discuss some pros and cons of using artificial intelligence during the writing process, and how its use can be reported. We hope that this practical guide to the structuring process will provide authors with a useful framework for the later addition of the full text and relevant references.

**Supplementary Information:**

The online version contains supplementary material available at https://doi.org/10.1186/s12998-026-00643-1.

## Introduction

Scientific articles that are easy to read and understand have some common traits: they are structured in a logical fashion, deal with one thing at a time, and keep to their intended topic. In contrast, articles that are difficult to read may have passages that are irrelevant or presented in an illogical order, and important concepts or arguments may be missing. Authors, reviewers, and readers will find that, after as little as two or three re-reads, they lose their ability to decode the main messages. Therefore, it is important to create an appropriate structure that includes all relevant items but has no superfluous material that crept in without being noticed.

Our professional experience as readers, writers, and supervisors has made us aware of some unwritten ‘rules’ that can facilitate the writing of well-structured and transparent articles. Our work as educators has shown us that there is a need for a practical instruction on how to become an efficient writer, with or without the help of artificial intelligence (AI). This is the first of three articles on how to structure the content of a scientific article with the aim of helping authors to write informative and easily read reports of clinical and epidemiological studies. The topic of this article is the Background section and the Research objectives. The second article deals with the Methods and Results section [1] and the third with the Discussion [2].

In the present article, we discuss the purpose of the Background section and explain how to build informative research objectives that form the basis for structuring the whole article. We then present an approach for planning the structure and contents of the Background section as preparation for writing the full text later. Finally, we discuss the possible uses of AI in preparing the Background section. We illustrate our text with examples selected from articles published in this journal, showing how other authors have approached the various parts of the Background section.

## What is the purpose of the Background section?

The Background section introduces the reader to the research topic and provides the rationale for the research objectives. Specifically, the reader expects to find the following:

- A presentation of the study topic, probably along with some information to show why it is relevant to research this topic,
- Sufficient and up-to-date information to enable readers to understand the context of the study,
- An indication of the specific knowledge that is missing about the topic to justify the stated research objectives, and
- A list of research objectives that indicate the study design and the type of results that will be shown.

Thus, the Background section informs the reader about the study topic and prepares the scene for the Methods section that follows. The text is often relatively short and typically has a narrative writing style so that it tells a story. The section should start by introducing or defining the study topic and then explain what we know and don’t yet know—leading into the study objectives.

Probably the simplest order of information in the Background section is:

- Identification/definition and/or justification of the study topic.
- Relevant factual details for the research objectives (e.g., a, b and c).
- The research gap(s) related to the research objectives (a, b, c).
- The list of research objectives (a, b, c).

However, it more often follows this model:

- Identification/definition and/or justification of the study topic.
- Relevant factual details for research objective (a) followed by research gap for (a).
- Relevant factual details for research objective (b) followed by research gap for (b).
- Relevant factual details for research objective (c) followed by research gap for (c).
- The list of research objectives (a, b, c).

Before writing your detailed text, check whether your target journal has any restrictions on the number of words in the Background section. If so, you may need to consider which details are the most important for your story.

## The importance of the research objectives

A manuscript that is inadequately structured and does not follow a clear path is difficult to read and understand. A serious problem is a mismatch between the research objectives as ‘advertised’ in the Background section and the findings reported in the Results section, i.e. either too much information or too little.

Your research objectives constitute your contract with the reader (what you promise to report on; nothing more and nothing less) and are, therefore, the most important aspect of your manuscript. In fact, the research objectives drive not only what you need to explain in the Background section but also the study design and, therefore, also the Methods. The results you will report and discuss also depend on the research objectives. If you consult your research objectives throughout the whole structuring process, they will help you to keep focus and produce a ‘tight’ manuscript without omissions and unnecessary text.

The research objectives typically appear at the end of the Background section in a published paper, but we deal with them early on in this article as they will help you structure the rest of your manuscript.

## Constructing the research objectives

Research objectives can be difficult to describe and often require many revisions. We suggest they are written jointly by the whole research team and as detailed as possible—and before starting data collection and report writing.

Here is a list of seven tips for constructing informative but stringent research objectives.

1. Either start with a general study aim followed by specific objectives, or start directly with a list of research objectives.
2. Select the term for your research objectives. (In this article, we use the words ‘research objective’. Terms that are often used instead are aim, purpose, research question, and null hypothesis.)
3. The research design should be evident from the research objective, either in the way the research objective is explained or through the use of a specific term.
4. State the condition/context or intended study population.
5. For each research objective, make it clear which type of analysis you will do and make sure that it can be answered. (Each research objective typically corresponds to one analysis or one set of analyses. Further, it must be possible to obtain a result or set of results for each research objective.)
6. Specify the ‘variable(s) of interest’ for each research objective (i.e. the variable(s) for which you are seeking answers and/or that will influence the main result of each analysis.)
7. Indicate any intervention and control groups.

When you have written your research objectives, they need formatting. Three things you need to think about are listed below.

1. If your text is a research protocol, your research objectives will be in the future tense. However, when you come to report on a completed study, you must write the research objectives in the past tense.
2. You can avoid repetition by placing frequently used words at the beginning (e.g., the study design, the verb that indicates what you did, or the condition you are studying).
3. Use a consistent style by not mixing research objectives (statements) with research questions (that require a question mark at the end).

For a detailed guide on constructing research objectives and examples from published articles, please see Additional file 1.

## A practical guide to structuring your Background section

When you have a set of detailed research objectives, it is easier to work out what information you need to present in the Background section and how to structure it. The following steps guide you through the process of structuring your Background section using subheadings and key words. If you do this before you start writing full sentences, you are more likely to end up with a relevant and logical text that justifies the research objectives.

### Identify the key words in your research objectives

Start by writing out your research objectives in full and then, for each of them, make a note of the key words, i.e. the condition or study population, any interventions, the variables needed for the analysis, etc. You need to list these now so that you can later check that they are all explained under the “relevant factual details” further on in the Background section.

### Identification/definition and/or justification of the study topic

Most Background sections start by identifying or defining the topic of the research, usually a disorder (e.g., shin splints or low back pain). This first paragraph often includes a brief general justification for the choice of the topic on an individual or societal level (e.g., the suffering that it causes, the large number of people affected, and/or the high costs). Some examples of how this can be written are shown in Table 1.

Note that if you are testing a procedure, validating a method, or doing something purely ‘technical’, this type of justification is usually not relevant, and the definition of the topic will be sufficient. This can also be the case in situations where the consequences are well known.

Decide what your study topic is, and then how you want to introduce it to the reader: will you start with a definition of the topic only, or would it be relevant to also point out the consequences of the condition for individuals and/or society? Whatever approach you choose, its purpose is to justify research into the topic (but does not explain the reasons for your research objectives).

**Table 1:** Examples of how the topic can be presented (by a definition and/or relevance)

Examples of how to justify the topic of the study	Text in the article
The topic is defined, and prevalence and human suffering are emphasized	“Back related leg pain (BRLP), also referred to as sciatica, is one of the most burdensome variations of the prevalent LBP conditions impacting 30–60% of those with LBP[3, 4]. BRLP is characterized by radiating pain originating from the lumbar spine and traveling into the proximal or distal lower extremity with or without neurologic signs [3, 4].” (Leininger, Evans et al. 2025)[3]
The topic is mentioned, and prevalence and societal costs are emphasized	“Low back pain (LBP) is a common symptom that imposes a significant socioeconomic burden, for example, high medical costs and work absences [3]” (Fu et al. 2025)[4]
The topic is mentioned, and societal costs and human suffering are emphasized	“Shoulder pain is an important medical issue with significant health-care costs and an extensive impact on the affected individuals’ well-being including absence from work and disability [3]“. (Naseri et al. 2024)[5]

### Relevant factual details for the research objectives

The next part of the Background section provides further information to help the reader understand the context of your study. Depending on the focus of your study, it may explain symptoms, treatment options, risk factors, causes, mechanisms, etc.—but only what is needed to understand the specific research objectives stated later in the paper. For example, if the study objective is to investigate the association between different types of footwear and the development of shin splints in marathon runners, it is unlikely that you need to describe in detail the epidemiology of shin splints in the general population, the various treatment options, or the history of marathon races.

Facts presented in this section should be accompanied by one or more relevant references, and these references should be placed next to the information that they support. The correct referencing method is shown in the following example: “Casual runners have a greater prevalence of foot pain (1) whereas marathon runners are more likely to report shin splints (2). These differences may be explained by natural selection (1,3), strengthening of the arch with long-term running (3,4), and/or differences in the definition of pain (5).” In the next example, the reader does not know which reference belongs to which statement: “Casual runners have a greater prevalence of foot pain whereas marathon runners are more likely to report shin splints (1,2), These differences may be explained by natural selection, strengthening of the arch with long-term running, and/or differences in the definition of pain (1,3–5)”. This second approach is not helpful for a reader wanting to find the specific reference for one of the pieces of information.

Note that if someone else publishes on your study topic while you are conducting your study, you use that information in the Discussion section—the Background section describes the situation before you conducted your own study.

Generally, all the key words that are present in the research objectives (e.g., the disease or population studied, any interventions, variables of interest) should have been at least mentioned in the background text. Thus, readers should not get any surprises when they come to the Research objectives!

Determine which aspects of the research objectives need explaining under this ‘relevant factual information’. You will need to define and describe the relevant concepts and ensure that you deal with all the key words you noted above in the research objectives (including the variables of interest).

As you structure your notes, keep in mind which aspect of the research objectives you are dealing with and make sure that you do not add any irrelevant text only because it is something you know about. The background text must not be written on a ‘nice-to-know’ basis, but on a ‘need-to-know’ basis.

### Identify main references

Each factual statement requires one or several suitable references. If you haven’t already done this under the steps above, select these references, check again that they are suitable, and make a note of them beside the factual information they support in the Background section.

### The research gap(s) related to the research objectives

After explaining relevant information about what we know about the study topic, you can now indicate what knowledge is still missing, i.e. the research gap(s). These should provide the rationale for your research objectives. There is usually one research gap for each of your research objectives. Examples of words that typically signal a research gap are however, but, nevertheless, has not been shown, poorly understood/researched, gaps, lack, and no information/studies.

In clinical and epidemiological studies, the research gaps can often be classified in four ways:

- ‘Jigsaw puzzle’: evidence exists in the area, but it is patchy with some links or steps missing. In this case, you do not need to explain the whole picture, just the area surrounding the missing information.
- ‘Ping-pong’: when the existing evidence is contradictory, with some studies finding a treatment to be useful while others find it is not, or when some studies identify a phenomenon as being a risk factor, but others fail to do so. Here, you can list the studies that go in one direction vs. those that go in the other direction, without too many details.
- ‘Hole’: occasionally, there is no previous evidence and little knowledge about the study topic (perhaps it is a new diagnosis or a new treatment). Here, you just need to write something like: “To our knowledge, no previous studies….”, “At the time of planning this study, we could not find any….”. If this lack of knowledge was identified in a (high-quality) systematic review, you can merely refer to that, without extra references. (“A recent systematic review (ref) concluded that no studies have dealt with this question”).
- ‘Clinical myth’: a clinical concept or procedure that is generally acknowledged as ‘the truth’ (usually based on long-term clinical use) but in fact has little supporting evidence in the literature (or perhaps no previous research at all). Here, you explain the case and state that you could find no supporting evidence. If you cannot find any references to how a procedure is used or how often etc., you could write something like “In our experience…”, “This method appears to be commonly used when…”.
- Some studies have no classifiable gaps, e.g., validation studies and pilot studies that are conducted to confirm whether procedures/tests are possible and trustable, and studies that are conducted on a technical advancement that can open new horizons or verify present knowledge.

Examples of how various gaps have been presented in the literature are found in Table 2.

**Table 2:** Examples of how research gaps can be presented in the Background section

Approaches	Text in the article
The words “although” and “poorly understood” catch the reader’s attention to a research gap in the form of a ‘jigsaw puzzle’	“…Although numerous studies have investigated the efficacy of manual therapy for NSLBP, the factors associated with clinical improvement following these interventions remain poorly understood [8].” (Barbier et al. 2025)[6]
The words “poor predictive accuracy” catch the reader’s attention to a research gap in the form of a ‘ping-pong’	“…Some [13, 20, 21] reported that SBT risk groups have moderate associations with pain and disability outcomes while others [11, 14, 22] found poor predictive accuracy.” (Fu et al. 2025)[4]
The words “however” and “lack” catch the reader’s attention to a research gap that possibly indicates a ‘hole’	“…However, there remains a notable lack of understanding regarding students’ confidence in applying SMT….” (Nim et al. 2025)[7]
The words “however” and “not investigated” catch the reader’s attention to a research gap in the form of a ‘hole’	“…However, the clinical implications of individually tailoring the content of manual intervention (such as SMT) have not been investigated.” (Galaasen Bakken et al. 2025)[8]
The word “gaps” catches the reader’s attention followed by a deduction based on previous factual details indicating a jigsaw puzzle	“…These gaps between ideal and actual treatments necessitate further investigation into how patients and providers understand, recommend, or adopt self-management strategies for BRLP.“ (Ziegler et al. 2025)[9]

For each of your research objectives, identify a research gap. Determine (for your own use) what sort of research gap it is (jigsaw puzzle, ping-pong, hole, myth) as this will help you to use your references appropriately.

Remember that the relevance of the topic and the research gaps cover two different aspects of the justification of your study. If the topic is irrelevant, it does not matter what sort of gaps you can identify; the study would not be worthwhile spending time and money on. Conversely, if the topic is relevant but there are no compelling gaps, the same still applies. The latter mistake may occur if the literature review omitted important previous research!

### Determining where to place the research gaps

If you have several research gaps, consider where it is most relevant to place them: should they come after all the factual details and placed before the research objectives, or should they be placed individually at different places within the factual details? If necessary, move your research gap(s) to a different location in the plan for your Background section, thinking about how best to relate them to the factual information you have already noted down.

### The full research objectives

The Background section ends with the research objectives, sometimes preceded by a few words that link them to the previous text, or perhaps starting with an overall aim or goal (Table 3).

**Table 3:** Examples of how research objectives can be linked to the previous Background section

Linking approach	Text in the article
A summary of the problem brings the reader to the research objectives	“…This underrepresentation highlights the need to better understand the characteristics and treatment outcomes of older patients who do seek chiropractic care. The aims of this study were …” (Hansen et al. 2025)[10]
The word ‘therefore’ links the previous arguments with the resulting research objectives	“…Therefore, the aim of this study was to investigate associations between….” (Nim et al. 2025)[7]
A brief rationale, including the general study approach, introduces the reader to the research objectives	“…To further expand the BCW knowledge base, our team undertook a theory-informed evaluation of BRLP self-management interventions. The purpose of our study was to identify barriers and facilitators to self-management for persons…” (Ziegler et al. 2025)[9]
The research objectives are preceded by a heading. No other link was needed. After a list of the research objectives, the authors inform the readers of the results they expect to obtain.	“Objectives and hypotheses This trial’s primary objective was to determine … We hypothesized that the patients who received ….” (Naseri et al. 2024)[5]

Move your full research objectives to the bottom of the text (removing your list of keywords—they were for your own use only), perhaps introducing them with a short text or a general aim.

If you later remove a research objective, either because you decide this before submitting the manuscript to a journal or in response to reviewers’ comments, make sure you also delete any of the relevant information and the corresponding research gap that have now become irrelevant. Similarly, if you add a research objective, you may need to add further factual details or another research gap. Therefore, keep a copy of your structured plan in case you need it later.

## Using AI for the Background section

The various aspects of the Background section can be planned or written with the aid of artificial intelligence (AI). The needs differ between those who are native English speakers and those who are not. Most of us use the spelling and grammar controls in our word processing programs, but there is a rapidly growing assortment of generative AI programs that can collect and synthesize information and create text on command. Several researchers have begun to discuss the advantages and disadvantages of using AI in academic writing, as in a recent systematic review [11].

Some positive take-home messages from that extensive review are that the use of AI can save time, create ideas, help in the search and synthesis of information, and produce audience-adapted texts. Negative aspects include biased and incomplete information searches, inadequate referencing, and production of inaccurate information (including invention of credible but non-existing information, “hallucinations”, and the omission of important nuances). Other concerns relate to legal issues: because a chatbot is not a legal entity, it cannot be made responsible for written text, and the risk of plagiarism arises due to AI collecting and synthesizing information from existing literature. The material that researchers ask the chatbot to work on will usually enter the public domain, so authors risk loss of ownership. To these concerns, we would add that the learning of skills and critical thinking are important aspects in scientific fields. Therefore, AI should be used as a support during the writing stage rather than replacing the author’s own deliberations over structure and content.

For example, after you have structured the Background section, listed the keywords for all relevant information, and found and placed relevant references alongside the factual information they support, you might find it helpful to ask AI to suggest a text. This approach would ensure that you retain control over the material that is included. The resulting text will likely be grammatically correct but possibly a bit bland.

A literature review of scientific journals by Ganjavi et al. [12] found a lack of guidelines and consensus on whether the use of AI in academic writing should be reported at all, and if so, how and where it should be reported. The review authors provide some valuable practical information for the research community. First, authors should find out if the journal they are aiming for allows for the use of some type of pretrained generative AI assistance. Thereafter, they should remember that authors, and not the AI, are responsible for any published text, and that the AI program cannot be one of the authors.

There are various places in the text of an article where you can report assistance from AI, e.g., in the Methods section or under ‘Acknowledgements’ or other headings after the main text, but also in a cover letter. Explain the type of AI used (name, model, version) and what you used it for, using guidance from the journal and/or the publisher. For example, Ganjavi et al. [12] state under their own additional information: “Generative artificial intelligence: ChatGPT 3.5 was used for the grammatical check of the introduction and discussion paragraphs. The authors validated the output and take full responsibility for the content.”

In September 2025, the International Association of Science, Technical & Medical Publishers (STM) published a classification framework for how authors might use generative AI in academic writing [13]. The STM framework lists nine approaches to using AI when preparing manuscripts and is a useful guide for authors when reporting how they have used AI.

## Summary

The Background section informs the reader about the study topic and prepares the scene for the Methods section that follows. It is usual to start the Background section by identifying the study topic and explaining why it is relevant to study. The text that follows presents relevant factual details about the topic, followed by the research gap or gaps that lead naturally on to the list of research objectives.

A ‘good’ Background section is one that includes only information relevant to the stated research objectives, makes a clear case for conducting the current study, and presents the research objectives in such a way that the reader can easily identify the key aspects, e.g., the study design and overall methodology, the study context, comparative groups, and the variables of interest.

In this article, we have provided a series of tips for writing clear and informative research objectives and present different ways of identifying research gaps to feed into these objectives. We explain how these gaps will guide the type and amount of factual information and how to link the background text to the list of research objectives.

Artificial intelligence should be used with care when structuring the content of the Background section as such texts need to be directly targeted to the stated research objectives. Furthermore, authors should not underestimate the benefits of learning and practising critical thinking skills when working out how to elegantly get from the study topic at the start of the section to the list of research objectives at the end!

## Electronic Supplementary Material

Below is the link to the electronic supplementary material.


Supplementary Material 1


## Data Availability

No datasets were generated or analysed during the current study.

## Declarations

### Use of artificial intelligence tools

AI (Word Editor) was used to suggest spelling and grammar improvements in the text.
