# Supplementary material for: Practical guide to structuring the Background section in clinical and epidemiological studies
Source: Chiropr Man Therap. 2026 Sep 1;34:43. doi: 10.1186/s12998-026-00643-1 (PMC13531733; doi:10.1186/s12998-026-00643-1)
Supplement: Supplementary file 1 [file 12998_2026_643_MOESM1_ESM.docx]

# Additional file 1. A guide for writing informative research objectives

Charlotte Leboeuf-Yde and Claire Gudex

## 1. Either start with a general study aim followed by specific objectives, or start directly with a list of research objectives

If you have a complicated research project, it might be helpful to start the research objectives with an introductory sentence about its general aim or purpose and continue with the specific research objectives. It could be written like this: “*The overall aim of this prospective observational study was to better understand the factors related to the development of shin splints in middle-aged marathon runners. Our specific research questions were: Do i) anatomical factors, ii) biomechanical factors, iii) ratio of training to rest, and iv) type of running surface show an association with the incidence of shin splints?”*

If the research objectives are easily understood without the introductory ‘general aim’ or ‘overall purpose’, then just present all the research objectives. This can be done in three ways: 1) They can be presented in bullet points, starting with *“Research objectives:”* giving each bullet point (and research objective) a separate line; 2) They can be written in an ordinary sentence, where the research objectives are listed following an introductory text, such as *“The research objectives of this prospective observational study were to determine whether anatomical factors, biomechanical factors …etc”*. This second option is the one that is most difficult for the reader to understand and remember; 3) Discretely numbered research objectives within the sentence make them easier to understand, as in “*The research objectives of this prospective observational study were to determine whether i) anatomical factors, ii) biomechanical factors… etc.”.*

Unless a description of the study population is one of your research objectives, there is no need to have a research objective that describes how you collected the data for this description (typically age, sex, socio-demographic factors, and/or clinical status shown in a ‘Table 1)’. However, if you will also use these variables in any of your analyses, they must be identified within the relevant research objective(s).

## 2. Select the term for your research objectives

In this article, we use the words ‘research objective’ to indicate what sort of answers we are looking for. Terms that are often used instead are *aim, purpose, research question*, and *null hypothesis*. Select which term you prefer and be consistent throughout your text. It does not matter which term you use, providing you use it appropriately. For example, a research question is phrased as a question and ends with a question mark, as in “*What is the difference between groups A and B in mean pain score after three months?*” This question can be worded as a research objective: *“We investigated the difference between groups A and B in mean pain score…*”. A null hypothesis assumes no significant difference or effect until proven otherwise by the study results. Examples are “… *that there will be no difference between groups A and B in mean pain score after three months*” or “… *that the exercise regime will have no effect on the Oswestry Disability Index score at 6-month follow-up”.* A ‘null hypothesis’ should not be confused with an ‘hypothesis’ as in ‘assumptions’ or ‘expected findings’, which are not synonymous with the statistical term ‘null hypothesis’ (which is now only rarely used in clinical studies).

When you have several research objectives, they can, of course, be referred to as ‘the first’, ‘the second’ etc., or as ‘the main’ and ‘additional’. However, be careful with the terms ‘primary’ and ‘secondary’ as they are reserved for studies in which a power calculation was done on one selected variable. In such cases, ‘primary objective’ refers to the research objective that includes this variable. When other variables are tested as well, their results do not carry the same weight, for which reason they are ‘secondary’.

## 3. The research design should be evident from the research objectives

A research objective can sometimes be dealt with using more than one research design. For example, “*The aim was to determine the anatomical and sociodemographic risk factors for the development of shin splints”*. The question is: Was this done in a predictive study or in a systematic review? Therefore, the choice of research design should be obvious within the text, typically by the inclusion of its name, as in this example: “*The aim of this prospective observational study was to determine anatomical and sociodemographic risk factors for the development of shin splints.”*

Alternatively, you can indicate the research design using words such as *describe, observe, follow, detect, compare, outcome, efficiency, and efficacy.* Example: “*The research objectives of this study were to describe the development of shin splints in marathon runners over a period of six months and to detect any anatomical and/or sociodemographic risk factors.”* This makes the reader understand that this was a prospective outcome study which tested associations. If *“self-reported”* was added, we would also understand that data were collected via e.g., questionnaires or a diary. Further details can be described in the Methods section.

## 4. State the condition/context or intended study population

Study results can differ depending on the study setting (e.g., hospital vs community-based clinic), the characteristics of the participants (e.g., age, sex, diagnosis, severity of disease, behaviour such as marathon runners vs recreational joggers), and perhaps the timing (e.g., follow-up of 3 months vs 2 years; summer vs winter; 2010-2015 vs 2020-2025). Therefore, the most relevant of this information should be stated in the research objectives to enable the reader to judge the results in the correct context or perhaps to compare with their own setting.

## 5. For each research objective, identify the analysis you will do and make sure that it can be answered

Each research objective typically corresponds to one analysis or one set of analyses. Further, it must be possible to obtain a result or set of results for each research objective.

An easy way to check if a research objective is expressed in such a way that it is possible to provide an answer, is to transform it into a research question**.** Example: “*We determined the prevalence of patients who expressed moderate or severe anxiety when scheduled for surgery*”. If presented as a research question, it could read: “*What was the proportion of patients who expressed moderate or severe anxiety when scheduled for surgery?”* The answer to this research objective or question could be: “67*%”.* In other words, this is a ‘workable’ research objective.

Be careful with words such as *observe* and *study* in your research objectives as they can be too vague and not lead to a clear answer or result. For example: "*The aim of this study was to observe patients during their stay in the surgical ward.”* This cannot be transformed into a research question, nor can it be easily answered (“*The study findings show that…it was possible to observe patients in the surgical ward”).* This is not a ‘workable’ research objective. We suggest you also do not choose *looked at,* because it is too superficial.

## 6. Indicate the main variable(s) of interest for each research objective

Each research objective needs to specify the ‘variable (or variables) of interest’, i.e. the variable(s) for which you are seeking answers, or which will influence the main result of each analysis.

These variables have specific uses in the study. They can be simply descriptive, as when you are searching for prevalence or incidence estimates. However, most studies test for associations between two or more variables. Thus, if a research question is worded like this: *“Is age positively associated with fear of surgery?*”, ‘age’ is the independent variable and ‘fear of surgery’ the dependent variable in the analysis. Any modifiers, confounders, and other extrinsic variables must also be apparent in the research objectives, as in this simple example: “*Is age positively associated with fear of surgery, taking into account the type and length of surgery?”*, which shows that both ‘type of surgery’ and ‘length of surgery’ were added to the analysis.

Variables can be quite diverse depending on your study topic (e.g., sociodemographic characteristics, symptoms, types of behaviour, clinical and laboratory test results, cell types, genes identified), and their description can be simple (if a single variable) or more complicated (if several variables).

An example of a single variable is: *“In this 12-month observational study, we determined the proportion of marathon runners with self-reported shin splints at any time over the study period.”* The variable of interest is ‘self-reported shin splints’, and you will obtain an estimate expressed as a percentage of all participants who reported to have had this.

Many research objectives deal with several variables, such as: “*In this 12-month observational study,* *the long-term outcome of shin splints was determined using a self-reported questionnaire enquiring about night pain, pain on walking, and use of pain medication”.* The variables of interest are ‘night pain’, ‘pain on walking’, and ‘use of pain medication’. However, these variables will need some further definitions, such as ‘night pain yes/no’ or ‘number of nights per week being woken by pain in the legs’. This can be specified in the Methods section to avoid reader-fatigue.

Another way of listing many variables is to use their ‘family names’, perhaps followed by some of the variables that fit under each of these. Example: *“In this 12-month observational study of marathon runners with shin splints, we determined the predictive value of various sociodemographic (e.g., age, sex, educational level) and anatomical factors (e.g., BMI, pes planus/pes cavus, genu valgum/genu varus)”*—again, leaving the details for the Methods section.

You can also discretely number the variables to make it easier for the reader to differentiate them in their minds. Example: *“In this 12-month observational study of marathon runners with shin splints, we determined the predictive value of i) various sociodemographic factors, ii) anatomical findings, and iii) self-reported training patterns”.*

| **Different ways to present the variables of interest** | **Text in the article** |
| --- | --- |
| One variable | …*to achieve this aim, we used ultrasound to observe and measure the separation of the spinous processes…* (Kruse et al. 2025) [1] |
| Several variables are presented under ‘family’ names, e.g., demographic (age, sex), anthropometric (height, weight) | … *Therefore, the aim of this study was to investigate associations between demographic, anthropometric, and SMT experience factors and confidence and competence in delivering specific SMST force-time characteristics on mannikins.* (Nim et al. 2025) [2] |
| ‘Family’ names are provided with examples of which variables they included | … *The objective was to assess feasibility using pre-specified targets for recruitment and enrollment (e.g., #s screened and enrolled/month), intervention delivery (e.g. #s attending minimum visits and satisfied with intervention) and data collection (e.g., #s completing weekly and monthly surveys).* (Leininger, Evans et al. 2025) [3] |

For some additional examples of how to deal with the variables in research objectives, see Additional Table 1.

**Additional Table 1.** Examples of how to report variables of interest in the research objectives

## 7. Indicate any intervention and control groups

Some study designs involve intervention and control groups, and the reader should be able to clearly see this from the research objectives. Otherwise, they might get an unwelcome surprise when they come to the Methods section and cannot understand why you are suddenly describing an intervention. Make it clear whether you are comparing one treatment to another or to a sham/placebo intervention. For example, “*The aim of this 3-month unblinded observational study was to compare frequency of self-reported shin pain among marathon runners aged 40-55 years wearing either customized and cushioned running shoes or standard running shoes”.* Here, the two shoe types could be considered the intervention and the control.

Finally, check the content of your research objectives using the following tips:

**Tips for checking your research objectives:**

- Make sure that all the key words for your study (e.g., design, population, comparisons) are mentioned in your research objectives (hint: look at your study title to see if you have forgotten anything!)
- Check that each of your research objectives relates to an analysis or a set of analyses (such as a univariate analysis followed by a multivariate analysis); usually there is only one type of analysis per research objective (e.g., correlations in the first research objective, regression analysis in the second).
- Think of all the answers you hope to obtain and make sure you have them all covered in your research objectives.
- When you are happy with your research objectives, write them as questions and try to imagine the answers you could get, i.e. ensure you have ‘workable’ research objectives.
- Ask someone else to read your research objectives (without giving them any other information) and see if they can tell you what the study was about (beware of too polite friends and colleagues!). Keep working on your research objectives until they are clear and complete.

1.	Kruse R, Gudavalli M, White B, Rider S, Greenwood D, Rogers C. Ultrasound measurements of lumbar spinous process movement during flexion distraction manipulation: a preliminary descriptive cross-sectional study with healthy participants. Chiropr Man Therap. 2025;33(1):29.

2.	Nim C, Smith N, Starmer D, Wang S, Choi G, Alayed A, et al. Chiropractic students' characteristics influencing confidence and competence in modulating spinal manipulation force-time characteristics of specific target forces: a secondary analysis of a cross-sectional study. Chiropr Man Therap. 2025;33(1):15.

3.	Leininger B, Evans R, Greco CM, Hanson L, Schulz C, Schneider M, et al. Supported biopsychosocial self-management for back-related leg pain: a randomized feasibility study integrating a whole person perspective. Chiropr Man Therap. 2025;33(1):6.
